# DNA Methylation May be Involved in the Analgesic Effect of Hyperbaric Oxygen via Regulating FUNDC1

**DOI:** 10.1155/2020/1528362

**Published:** 2020-02-18

**Authors:** Kun Liu, Hao Wu, Rui Gao, Guang Han

**Affiliations:** Department of Anesthesiology, Shengjing Hospital of China Medical University, Shenyang, China

## Abstract

**Background:**

Neuropathic pain (NP) is a type of chronic pain which lacks predictable, effective, and safe therapeutic options. We investigated the role of hyperbaric oxygen (HBO) in expression of FUN14 domain-containing 1 (FUNDC1), which is associated with DNA methylation.

**Methods:**

We randomly divided rats into four groups: sham operation (S), S + HBO, chronic constriction injury (CCI), and CCI + HBO. Lumbar (L)4 and L5 dorsal root ganglia (DRGs) were used to assess expression of DNA methyltransferase (DNMT)1, DNMT3a, and DNMT3b by western blotting and RT-PCR. Pain-related behaviors were evaluated using mechanical withdrawal threshold and thermal withdrawal latency analysis. Western blotting was also used to assess expression of FUNDC1, BCL2, and adenovirus E1B19 kDa-interacting protein 3-like (NIX) and BCL2 and adenovirus E1B19 kDa-interacting protein3 (BNIP3). And we also examined the changes of FUNDC1 with immunofluorescence. Nonnucleoside DNA methyltransferase inhibitor RG108 was administered prior to CCI. The pain-related behavior and western blotting changes were examined in all groups.

**Results:**

DNMT3a expression was higher on day 14 after CCI. HBO downregulated DNMT3a mRNA and protein expression, but not those of DNMT1 and DNMT3b. HBO increased pain-related behavior significantly, while it was down-regulated by RG108. In HBO groups, FUNDC1, NIX, and BNIP3 expression was upregulated more significantly than in the CCI group. In addition, FUNDC1 protein colocalized with NeuN and rarely with glutamine synthetase. However, expression was reduced when RG108 was administered. Immunofluorescence showed that FUNDC1 was upregulated after HBO treatment.

**Conclusion:**

Our findings suggest that DNA methylation is involved in the analgesic effect of HBO via the regulation of FUNDC1.

## 1. Introduction

Neuropathic pain (NP) is a type of chronic pain that lacks predictable, effective, and safe therapeutic options. In the past few years, there has been extensive research into novel treatments for NP. Nevertheless, the number of NP patients continues to increase and treatment remains costly and time consuming [1]. Hyperbaric oxygen (HBO) is a noninvasive treatment that has been widely investigated and can relieve NP [2]. Our previous research discovered that the alleviating effect of HBO on NP is associated with mitophagy [3]; however, the detailed mechanisms of how mitophagy alleviates pain are not fully understood.

In the nervous system, mitophagy often has a protective effect on nerve damage or apoptosis [4]. FUN14 domain-containing 1 (FUNDC1), as a mitochondrial membrane protein, is associated with mitophagy and is involved in ischemia-reperfusion (I/R) injury [5]. However, the functional roles of FUNDC1 in NP remain largely unclear. Some research has found that FUNDC1 increases mitophagy in reperfused tissue and prevents apoptosis [6]. In addition, FUNDC1-related mitophagy also attenuated oxidative stress in nerves, alleviated mitochondrial damage, promoted mitochondrial biosynthesis, maintained ATP production, and sent prosurvival signals for I/R injury [7]. Several other studies had found that FUNDC1-related mitophagy is inactivated by reperfusion [8]. Therefore, we wanted to explore the upstream molecular signals that control FUNDC1-mediated mitophagy in I/R injury. Although previous studies had suggested that NP is primarily regulated by methylation of DNA methyltransferase 3a (DNMT3a), the role of DMNT3a methylation in nervous I/R injury has not been fully elucidated [9].

DNA methylation is an important modification method in regulating mitophagic processes. The patterns of DNA methylation are established and maintained by DNMTs [10]. Recent research has discovered that DMNTs catalyze the transfer of a methyl group from S-adenosyl-methionine to cytosine residues in gene regulation, which leads to chromatin remodeling [11]. It has also been shown that DMNT3a is mainly involved in the establishment of DNA methylation in the spinal cord [12]. And it functioned in synaptic plasticity, memory formation, and behavioral plasticity [13]. Since some of the pain process is related to synaptic plasticity, it is likely that DNA methylation regulates the development of NP.

HBO which is beneficial for formation of new blood vessels and repairing the function of nerve tissue is a noninvasive treatment for I/R disease [14]. Some researchers have reported that preconditioning HBO can protect against nervous I/R injury [15]. In our previous research, we discovered that HBO therapy palliates chronic constriction injury- (CCI-) induced NP in rats by upregulating mitophagy. In our preliminary experiments, we found that HBO inhibited DNMT3a and enhanced FUNDC1 expression. In the present study, we hypothesized that HBO would alleviate NP by involvement of DNMT3a methylation and mitophagy.

## 2. Materials and Methods

### 2.1. Materials

The following materials were used: ECL western blotting kit (Solarbio, Beijing, China), horseradish peroxidase conjugated rabbit anti-goat IgG, goat anti-rat IgG (Pierce, Rockford, IL, USA), rabbit anti-rat FUNDC1 (ab224722, Abcam, Cambridge, UK), rabbit anti-rat RG108 (ab141013, Abcam), mouse anti-rat DNMT1 (ab13537, Abcam), rabbit anti-rat DNMT3a (ab4897, Abcam), rabbit anti-rat DNMT3b (ab2851, Abcam), rabbit anti-rat NIX, BNIP3 IgG (D4R4B, CST, Danvers, MA, USA), anti-NeuN (1 : 100, Temecula), mouse antiglutamine synthetase (1 : 1000, Sigma), fluorescence microscope X81 (Olympus, Tokyo, Japan), total protein extraction kit (Keygen Biotech, Nanjing, China), and transmission electron microscope H-600 (Hitachi, Japan).

### 2.2. Animals

Male Sprague-Dawley rats (260 ± 20 g) were purchased from the Animal Center in Shengjing Hospital of China Medical University (Shenyang, China). The Ethics Committee of the Center for Scientific Research with Animal Models at Shengjing Hospital of China Medical University (Shenyang, China) approved the experiments, which were performed following the National Institutes of Health guidelines. A total of 48 rats were randomly separated into a normal group without treatment (*n* = 24) and a DNMT inhibitor group treated by intrathecal injection of nonnucleoside DNA methyltransferase inhibitor, RG108 (*n* = 24). Each group included four subgroups (*n* = 6 rats per group for three repeats) as follows: a sham operation group (S) in which the sciatic nerve was exposed, but not ligated, and without HBO treatment; sham operation plus HBO group (S + HBO) in which the sciatic nerve was exposed and HBO treatment was administered; chronic sciatic nerve injury group (CCI) in which the sciatic nerve was ligated without HBO treatment; and CCI plus HBO treatment group (CCI + HBO) in which HBO was initiated 6 h after CCI once daily for the following five days.

### 2.3. Experimental Process

**Figure 1:**
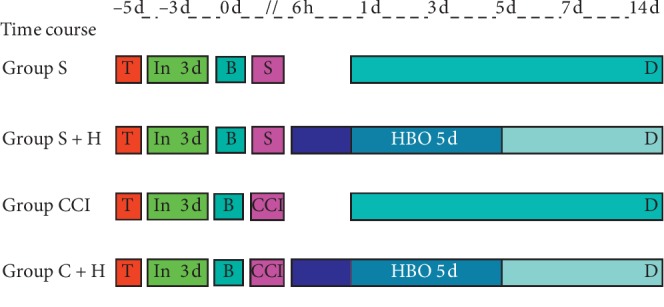
Experimental process. This course is processed in the *N* normal group or in the RG108 group. In 3 d: inject DMSO/RG108 for the 3-day group; B: baseline values; S: sham operation; CCI: chronic constrictive injury; d: day; D: death sample taken; HBO: hyperbaric oxygenation; T: intrathecal cathetering; S: sham group; S + H: sham plus HBO group; CCI: CCI group; C + H: CCI plus HBO group; RG108: non-nucleoside DNA methyltransferase inhibitor; −5 d: 5 days before CCI (beginning point of intrathecal cathetering) −3 d: 3 days before CCI (beginning point of drug injection) 6 h: 6 hours after CCI (beginning point of HBO); 1 d, 3 d, 5 d, 7 d, and 14 d: 1, 3, 5, 7, and 14 days after CCI (time points of behavior test). Light blue: behavior test score; pink: surgery; dark blue: HBO treatment; green: injection of DMSO/RG108.

An intrathecal tube was implemented 5 days before CCI. The rats were observed in the following 48 h to confirm that there were no adverse reactions. We calculated the dosage (50 mg/kg) of RG108, dissolved in 0.1 ml dimethyl sulfoxide (DMSO), and injected into the epidural cavity once daily. Sterile DMSO (0.01%, 0.1 ml) or RG108 (50 mg/kg, 0.1 ml) was injected in the normal control or the DMNT inhibitor group, respectively, 3 days before intrathecal CCI. After three days of consecutive intrathecal injection, baseline mechanical withdrawal thresholds (MWTs) and thermal withdrawal latencies (TWLs) were measured in all rats 1 day before CCI. CCI or sham operation was performed in the morning. Pain-related behavior was observed 1, 3, 5, 7, and 14 days after CCI between 09:00 and 11:00 h each day. Rats in the CCI + HBO group were first exposed to HBO 6h after CCI once daily for the following five days. Finally, the rats were sacrificed and the L4 and L5 dorsal root ganglia (DRGs) were collected on day 14 after CCI (Figure 1).

### 2.4. Intrathecal Catheterization

Anesthesia was induced with inhaled 2.5% isoflurane before surgery and maintained with 2% isoflurane during surgery. After anesthesia, the rats were subjected to intrathecal catheterization. After location of L6 by the iliac crest, a longitudinal skin incision was made to expose the muscle and fascia. After fully exposing the L3-4 interspace, we penetrated the yellow ligament with a fine epidural catheter slowly and gently and then placed the catheter to the cephalic side by 2 cm. Colorless cerebrospinal fluid flowing out of the catheter confirmed the success of intrathecal catheterization. We fixed the catheter on the back of the neck to prevent it being scratched off by the rats. We injected 15,000 U penicillin intraperitoneally to prevent infection. After surgery, we observed the bilateral hind limbs and tail movements to confirm that there was no spinal nerve injury for 48 h.

### 2.5. CCI Model Preparation

Anesthesia was induced with inhaled 2.5% isoflurane and maintained with 2% isoflurane. A posterolateral incision was made on the right hind limb, and the right sciatic nerve trunk was found. We loosely ligated it with 4-0 silk thread to produce slight pressure on the epineurium. The momentary muscle contraction confirmed the success of the CCI model. Finally, the incisions were closed. Rats were returned to the cages after regaining consciousness. In the S and S + HBO groups, the sciatic nerve was only exposed without ligation.

### 2.6. HBO Treatment

Rats in the S + HBO and CCI + HBO groups were treated with HBO five times once daily following CCI. All the other rats were placed in the HBO chamber without high pressure and high oxygen concentration. On day 1, HBO was applied 6 h after CCI. HBO conditions were as follows: the HBO chamber was first purified with 90% pure oxygen, which was allowed to fill the chamber for 10 min. The high pressure was increased at a rate of 0.0125 MPa/min to 0.25 MPa/min for 60 min and subsequently decreased to normal pressure for 30 min at a constant rate. The rats in other groups simulated the environment of the HBO chamber without high oxygen and pressure.

### 2.7. Sample Collection

On day 14, rats were anesthetized with inhaled 2.5% isoflurane and a tube was inserted from the left ventricle to the ascending aorta. Saline (0.9%) was poured in until no red perfusate was released. After perfusion with 0.1 M phosphate buffer (pH 7.4), the L4 and L5 DRGs were removed.

### 2.8. Pain-Related Behaviors

All the rats adapted to the environment before behavioral testing. We carried out two kinds of behavioral tests (mechanical and thermal heat tests) at 1h intervals. The rats were observed at 09:00 and 11:00 h on days 1, 3, 5, 7, and 14 after CCI. MWT was tested with von Frey filaments. Rats were placed individually in a chamber and acclimatized to the environment for 30 min. We chose optimal von Frey filaments that were applied to the plantar surface of the hind paw for 6–8 s to observe the hind paw withdrawal response. TWL, to assess thermal sensitivity quantitatively, was measured by a mobile radiant heat source focusing on the hind paw of each rat. Rats were placed on the glass surface of a thermal testing apparatus and acclimatized for 30 min before testing. The paw TWL was the mean of three trials and recorded three times by a timer. We used 20 s as a cutoff to prevent potential tissue damage.

### 2.9. Western Blot Analysis

To obtain sufficient proteins, the ipsilateral L4 and L5 DRGs from rats were put together. The tissues were homogenized and ultrasonicated in chilled lysis buffer. Approximately, 10% of the homogenates in volume were used for total proteins. The total protein was separated with a total protein extraction kit according to the instructions. The remaining was centrifuged at 4°C for 15 min at 1,000 g. The supernatant was collected for cytosolic proteins and the pellet for nuclear proteins. Protein content was determined by diluting the protein with PBS to the same concentration. A total of 20 *μ*g protein was loaded for electrophoresis. SDS-PAGE was performed. After electrophoresis, gels were transferred to a cellulose membrane and immersed in 5% nonfat milk for 60 min. The membranes were incubated overnight with anti-NIX and anti-BNIP3 primary antibody with the dilution 1 : 250; anti-FUNDC1, anti-DMNT1, anti-DMNT3a, and anti-DMNT3b primary antibody with the dilution 1 : 200; anti-*β*-actin and anti-H3 primary antibody with dilution 1 : 200. *β*-Actin and H3 were served as an internal reference. Membranes were washed with PBS and incubated with a secondary antibody (1 : 1000) for 30 min, followed by rinsing with PBS. Expression of NIX, BNIP3, FUNDC1, DMNT1, DMNT3a, and DMNT3b was detected with the ECL western blotting substrate kit (Abcam, ab65623, UK) using Quantity One software on a Doc™ XR gel imaging system (Bio-Rad, Hercules, CA, USA).

### 2.10. Quantitative RT-PCR

Total RNA extraction and quantitative reverse-transcribed PCR were measured as follows: ipsilateral L4 and L5 DRGs from rats were collected together to obtain sufficient RNA. Total RNA was extracted using the miRNeasy mini kit (QIAGEN, Germany) and reverse-transcribed using ThermoScript Reverse Transcriptase (Baiaolaibo, Beijing, China). Template (1 *μ*l) was amplified with the Bio-Rad CFX96 real-time PCR system using specific primers. Each sample was run in triplicate in a 20-*μ*l reactive volume containing 250 nM forward and reverse primers, 10 *μ*l Advanced Universal SYBR Green Supermix (Bio-Rad), and 20 ng cDNA. The PCR amplification comprised 30 s at 95°C, 30 s at 60°C, 30 s at 72°C, and 5 min at 72°C for 39 cycles. We calculated the ratios of ipsilateral mRNA levels to contralateral mRNA levels using the 2^−△△Ct^ method after normalization to *Tuba-1a*.

### 2.11. Immunofluorescence

We dissected L4 and L5 DRGs on the ipsilateral side on day 14 after CCI. The separated DRGs were fixed in 2.5% glutaraldehyde and placed in the refrigerator for 12 h at 4°C before observation under the microscope. Samples were washed with PBS, fixed with osmic acid, dehydrated with acetone, and embedded with the resin. Finally, the samples were cut into thin sheets and observed by fluorescence microscopy. To determine FUNDC1 expression, the samples were subjected to immunofluorescence using rabbit anti-FUNDC1 antibody, which was observed under an inverted fluorescence microscope (X81, Olympus, Tokyo, Japan).

### 2.12. Statistical Analysis

Data analysis was performed using SPSS version 21.0 (IBM Corporation, Armonk, NY, USA). Data are expressed as mean ± SD. Two-way ANOVA was used to compare the indexes between groups. Differences were considered statistically significant at *P* < 0.05.

## 3. Results

### 3.1. DNMT3a Expression Is Increased in the Ipsilateral DRG after CCI

**Figure 2:**
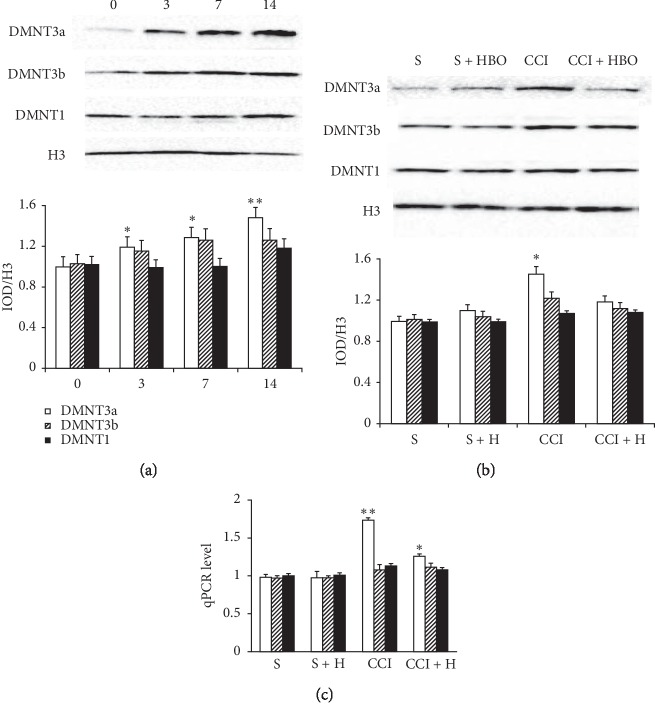
Qualitative and quantitative western blot and mRNA expressive levels of DNMT1, DNMT3a, and DNMT3b after treatment with S, S + HBO, CCI, and CCI + HBO groups.(a) DNMT3a protein showed time-dependent increased expression with the highest point on the 14th day in L4 and L5 DRG, but not DNMT1 and DNMT3b (*n* = 6 rats per group for three repeats, ^*∗*^presented compared with the S group, *P* < 0.05). (b) Western blot and qt-PCR also presented that DNMT3a became gradually stronger after CCI. And the protein and mRNA expression of DNMT3a in the CCI group was stronger than that in the CCI + HBO group (*n* = 6 rats per group for three repeats, ^*∗*^presented compared with the S group, *P* < 0.05). (c) Western blot in the CCI group is stronger than that in the CCI + HBO group (^*∗*^presented compared with the S group, *P* < 0.05). Qualitative and quantitative protein and mRNA expression of DNMT1 and DNMT3b after treatment in all groups had no significant difference (*n* = 6 rats per group for three repeats, *P* < 0.05).

In our previous study, we verified that HBO can upregulate mitophagy. However, we do not know whether the mechanisms are associated with DNMTs [3]. We evaluated expression of DNMTs by western blotting and RT-PCR in rats. We first examined DNMT expression in DRGs after CCI and sham surgery. CCI time dependently increased expression of DNMT3a protein in the ipsilateral L4 and L5 DRGs (Figure 2(a)). DNMT3a protein in the ipsilateral L4 and L5 DRGs increased by 1.2 fold on day 3 after CCI, 1.3 fold on day 7 after CCI, and 1.5 fold on day 14 after CCI compared to the corresponding sham DRGs (*P* < 0.05, Figure 2(a)). There was no significant time-dependent difference in DNMT1 and DNMT3b (*P* > 0.05, Figure 2(a)). As expected, expression of DNMT1, DNMT3a, and DNMT3b protein was analyzed using ipsilateral L4 and L5 DRGs on day 14 after CCI. Compared with the S and S + HBO groups, DNMT3a expression was higher in the CCI group but not in the CCI + HBO group (*P* < 0.05, Figure 2(b)). Expression of DNMT1 and DNMT3b did not change significantly among the groups (*P* > 0.05, Figure 2(b)). DNMT mRNA expression in the ipsilateral L4 and L5 DRGs during the observation period showed similar results. DNMT3a mRNA levels were higher in the CCI groups (*P* < 0.05, Figure 2(c)). However, expression of DNMT1 and DNMT3b in the ipsilateral L4 and L5 DRGs did not change significantly among the groups (*P* > 0.05, Figure 2(c)).

### 3.2. FUNDC1 May Be Associated with Analgesic Effect of HBO

**Figure 3:**
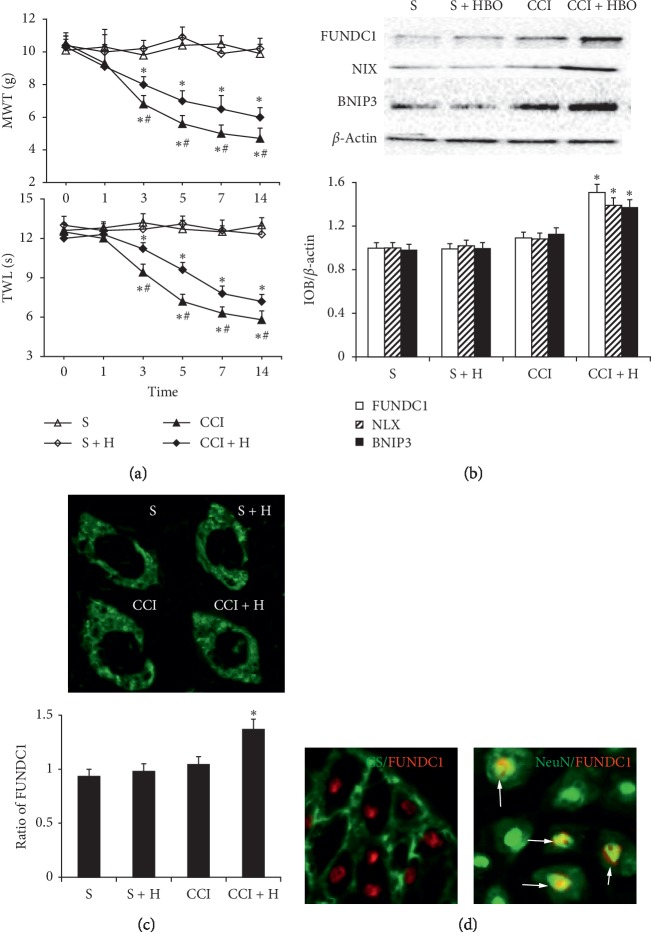
Mechanical withdrawal threshold (MWT) and thermal withdrawal latency (TWL) in each group (*n* = 6 rats per group). (a) The value of MWT and TWL decreased on the 3rd, 5th, 7th, and 14th day gradually after CCI which decreased mostly in the CCI group than in the CCI + HBO group (*n* = 6 rats per group for three repeats, ^*∗*^presented compared with the S group, *P* < 0.05). And the value of MWT and TWL in the CCI + HBO group was higher than that in the CCI group (# presented compared with the CCI group, *P* < 0.05).(b) FUNDC1, NIX, and BNIP3 expression was higher in the CCI + HBO group (^*∗*^presented compared with the S, S + HBO, and CCI groups, *P* < 0.05). (c) Compared with the S, S + HBO, and CCI groups, there were a large number of green fluorescence signals in the CCI + HBO groups on expression of FUNDC1 (^*∗*^*P* < 0.05).(d) The result showed that FUNDC1 coexpressed with NeuN in cellular nuclei and was not detected in the cellular nuclei of GS-labelled cells.

Tactile hypersensitivity determined by von Frey filaments and heat stimulation developed on the days 1, 3, 5, 7, and 14 after CCI. MWT and TWL scores decreased dramatically from day 3 to 14 in the CCI and CCI + HBO groups (*P* < 0.05, compared with the S and S + HBO groups, Figure 3(a)). This time-dependent change was similar to expression of DMNT3a on the corresponding days. MWT and TWL scores were higher in the CCI + HBO group, which indicated that HBO alleviated hypersensitivity (*P* < 0.05, compared with the CCI group, Figure 3(a)).

In our previous study, we verified that HBO can upregulate autophagy and mitophagy in nervous cells by affecting expression of LC3, P62, BNIP3, and NIX. Here, we evaluated the change in FUNDC1 expression by western blotting in rats that underwent CCI or sham operation. Compared with the S, S + HBO, and CCI groups, FUNDC1, BNIP3, and NIX expression was higher in the CCI + HBO group (*P* < 0.05, Figure 3(b)). To determine whether mitophagy was associated with FUNDC1, we observed expression of FUNDC1 by fluorescence microscopy, which has been shown to be an effective method of detecting localization. We immunostained all the groups with FUNDC1 and compared with the S, S + HBO, and CCI groups, and there was a large green fluorescence signal in the CCI + HBO group, showing expression of FUNDC1 (*P* < 0.05, Figure 3(c)). This indicated that HBO increased expression of FUNDC1 after CCI. We also examined the distribution pattern of FUNDC1 in the DRG. We found that FUNDC1 protein colocalized with NeuN and rarely with glutamine synthetase (a marker for satellite glial cells) (Figure 3(d)).

### 3.3. Blocking DNMT3a May Attenuate CCI-Induced NP and Decrease HBO-Associated FUNDC1 Expression

**Figure 4:**
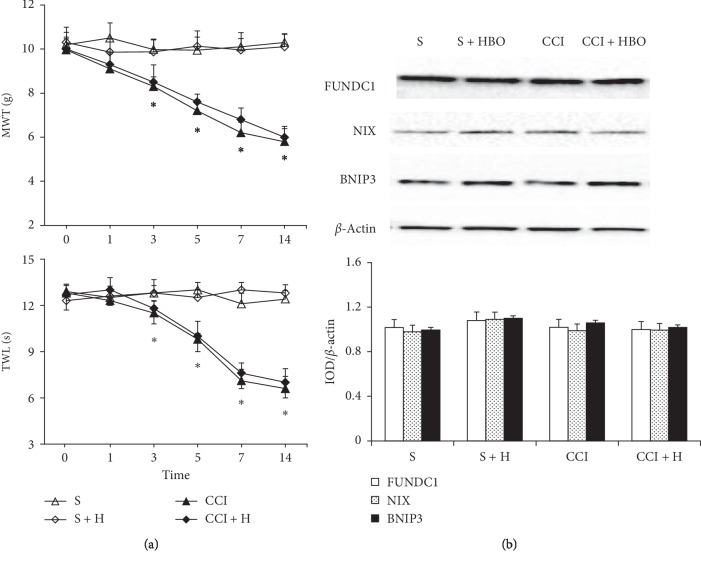
(a) MWT and TWL scores were presented in each group after RG108 administration (*n* = 6 rats per group). We could see the value of MWT and TWL also decreased on the 3rd, 5th, 7th, and 14th day gradually after CCI (*n* = 6 rats per group for three repeats, ^*∗*^presented compared with the S group, *P* < 0.05). But the value of MWT and TWL in the CCI + HBO group was similar to the value in the CCI group (*P* < 0.05). (b) FUNDC1, NIX, and BNIP3 expression were similar in all groups after DNMT inhibitor RG108 administration (*n* = 6 rats per group for three repeats, *P* < 0.05).

To verify that the expression of DNMT3a was associated with NP and FUNDC1, we treated rats with an isoform-nonspecific DNMT inhibitor (RG108) 3 days before surgery. MWT and TWL were also detected on days 1, 3, 5, 7, and 14 after CCI. MWT and TWL scores decreased dramatically from day 1 to 14 in the CCI and CCI + HBO groups (*P* < 0.05, compared with the S and S + HBO groups, Figure 4(a)). However, we observed that mechanical and thermal hyperalgesia were consistently attenuated in RG108-treated CCI rats after CCI surgery. The MWT and TWL scores became similar in the CCI + HBO and CCI groups, which indicated that the analgesic effect of HBO was reduced (*P* > 0.05, Figure 4(a)). After treatment with RG108, we evaluated the change in FUNDC1 expression levels by western blotting in rats that underwent CCI or sham operation. Expression of FUNDC1, BNIP3, and NIX was similar in all groups (*P* > 0.05, Figure 4(b)). The high expression of FUNDC1, NIX, and BNIP3 in the CCI + HBO group was reversed after RG108 administration, which indicated that upregulation of mitophagy by HBO had disappeared.

## 4. Discussion

Our previous study showed that the analgesic effect of HBO on NP is associated with mitophagy in rats [3]. In the present study, we investigated the molecular mechanism of the mitophagic effect of HBO. Why did we investigate DNA methylation? Firstly, DNA methylation is a key epigenetic mechanism which controls DNA accessibility and gene expression [16]. Some researchers have reported that blocking DNA methylation significantly affects pain-associated behavior tests in neuropathic and inflammatory pain [17]. Secondly, DNA methylation levels in some regions are suggested to play an important role in modulating replication or transcription of mitochondrial DNA since nearly the entire mitochondrial genome transcribes from this region [18]. DNA methylation affects NP as well as mitophagy. We were eager to explore the role of DNA methylation in HBO-associated mitophagy. Therefore, in this study, we sought to investigate the role of HBO in modulating DNA methylation and possible target enzymes contributing to NP. Finally, DNA methylation is a hot research topic at present. However, few studies have investigated its mechanism of action in NP. The role of DNMT is a major concern at present, but its mechanism in NP is unclear.

DNA methylation, especially CpG dinucleotides, is a regular epigenetic modification used in regulating nuclear gene expression [19]. Cytosine methylation signals can remodel the chromatin and downregulate gene expression that is mediated by a family of DNMTs [20]. DNMTs have three principle isoforms (DNMT1, DNMT3a, and DNMT3b) that establish and maintain patterns of DNA methylation in mammals [21]. Generally, DNMT1, a most abundant isoform in proliferating cells, could maintain the methyltransferase and copy methylation targets with replication forks during replication at sites of DNA repair. DNMT3a and DNMT3b methylate specific DNA targets in response to environmental factors [22, 23]. It has been observed that persistent pain can alter expression of DNMTs. It is reported that treatment that promoted DNA demethylation might have resulted in mechanical and thermal antinociception in a mouse oral cancer model [24]. Furthermore, DNA methylation regulates several additional pain and analgesia-related genes in various pain models [25]. Similarly, in our study, CCI increased DNA methylation in rats and time-dependently increased expression of DNMT3a protein in ipsilateral L4 and L5 DRGs, especially on day 14. However, there was no significant time-dependent difference in DNMT1 and DNMT3b. Compared with the CCI + HBO group, DNMT3a mRNA and protein expression was higher in the CCI group. However, expression of DNMT1 and DNMT3b did not change significantly among the groups. The results indicated that the analgesic effect induced by HBO was related to DNMT3a. So, we presume that DNMT3a methylated specific downstream DNA targets. In addition, the level of DNA methylation is also controlled by ten-eleven translocation methylcytosine dioxygenases (TETs), which causes oxidation of methylated DNA, resulting in demethylation. Due to the time and expense of the experiment, we did not conduct further research on the methylation targets and TETs. In future, we will explore the methylation targets of DNMT3a when HBO acts through mitophagy. And, we will test whether hyperbaric oxygen could influence the expression of TET1, TET2, and TET3.

Intrathecal injection of DNMT inhibitor RG108 reversed the upregulation of DNMT3a and simultaneously attenuated CCI-induced mechanical allodynia and thermal hyperalgesia. There was no significant decrease in thermal sensitivity in the RG108-treated CCI rats. To investigate further the involvement of DNMT3a methylation in NP, we determined the relationship between expression of FUNDC1 and thermal hypersensitivity. Our results showed upregulation of MWT and TWL in the CCI group after inhibition by RG108. In addition, there was no significant difference between the CCI and CCI + HBO groups in the pain behavior test. The expression of NIX, BNIP3, and FUNDC1 was also not significantly different between the CCI and CCI + HBO groups. We next investigated whether DNA demethylation reversed FUNDC1 protein expression correlated with thermal hyperalgesia following nerve injury. DNMT3a expression was almost completely suppressed in RG108-treated CCI and sham-operated rats. Intrathecal RG108 inhibited immunostaining of FUNDC1.

The key molecular mechanism to control mitochondrial homeostasis appropriately is mitophagy (a selective form of autophagy). In the past few decades, NIX, BNIP3, and FUNDC1 have been identified and characterized as essential factors for mitophagy although they might play different roles in modulating mitochondrial homeostasis [26]. In I/R injury, FUNDC1 exerts its protective action by reducing mitochondrial oxidative stress, blocking mitochondrial fission, attenuating mitochondrial calcium overload, promoting mitochondrial energy production, and preventing mitochondrial apoptosis [5]. Moreover, FUNDC1-related mitophagy can reverse ATP production, stabilize mitochondrial membrane potential, and block I/R-activated mitochondrial apoptosis [6]. Unfortunately, FUNDC1-related mitophagy is prone to be repressed by reperfusion injury and the upstream mediator has not been elucidated [27]. In the present study, we found that I/R injury and mitophagy activity are primarily regulated by DNA methylation. Inhibition of DNMT3a reverts FUNDC1 level to normal and therefore enhances protective mitophagy in DRGs. Our data confirmed that DNMT3a can be considered as the upstream regulator of FUNDC1-related mitophagy in a CCI model. In addition, DNMTs have also been found to be associated with other mitophagy mediators. The evidence suggests that DNMT3a effectively manages mitophagy by controlling expression of multiple mitophagy receptors, which firmly establishes a central role of DNMT3a in mitophagy modification.

Clinically, HBO reduces I/R-induced injury. However, the mechanism remains unclear. It is likely that mitophagy is related to the ability of HBO to enhance antioxidant enzyme activity and reduce edema in the nervous tissue [28]. Moreover, HBO promotes capillary regeneration and improves microcirculation. Our previous study tested HBO in rats with induced mitophagy in NP [3]. As expected, HBO reduced the pain behavior test and maintained nerve function because HBO increased mitophagy, which was significantly related to the dephosphorylated LC3-interacting region (LIR) motif of FUNDC1.

In summary, our results demonstrate that DNMT3a may participate in the pain process. There were several limitations to this study. Firstly, we tested the time points after CCI and chose an optimal time for mitophagy from day 1 to 14. However, the formation of NP was a long process. Adding days 21 and 28 after CCI may be more credible. Secondly, using FUNDC1 and DNMT3a antibody to colocalize and monitor this change may provide more persuasive results. Finally, if we could explore specific downstream DNA targets that are methylated by DNMT3a, we might gain deeper insight into the neuroprotective mechanisms of HBO mediated through mitophagy.

## Data Availability

The data used to support the findings of this study are included within the article.
